# Age-Specific Characteristics and Coupling of Cerebral Arterial Inflow and Cerebrospinal Fluid Dynamics

**DOI:** 10.1371/journal.pone.0037502

**Published:** 2012-05-30

**Authors:** Marianne Schmid Daners, Verena Knobloch, Michaela Soellinger, Peter Boesiger, Burkhardt Seifert, Lino Guzzella, Vartan Kurtcuoglu

**Affiliations:** 1 Institute for Dynamic Systems and Control, Department of Mechanical and Process Engineering, ETH Zurich, Zurich, Switzerland; 2 Institute for Biomedical Engineering, University and ETH Zurich, Zurich, Switzerland; 3 Neuroimaging Research Unit, Department of Neurology, Medical University Graz, Graz, Austria; 4 Institute of Social and Preventive Medicine, University of Zurich, Zurich, Switzerland; 5 Laboratory of Thermodynamics in Emerging Technologies, Department of Mechanical and Process Engineering, ETH Zurich, Zurich, Switzerland; University of Maryland, College Park, United States of America

## Abstract

The objective of this work is to quantify age-related differences in the characteristics and coupling of cerebral arterial inflow and cerebrospinal fluid (CSF) dynamics. To this end, 3T phase-contrast magnetic resonance imaging blood and CSF flow data of eleven young (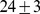 years) and eleven elderly subjects (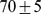 years) with a comparable sex-ratio were acquired. Flow waveforms and their frequency composition, transfer functions from blood to CSF flows and cross-correlations were analyzed. The magnitudes of the frequency components of CSF flow in the aqueduct differ significantly between the two age groups, as do the frequency components of the cervical spinal CSF and the arterial flows. The males' aqueductal CSF stroke volumes and average flow rates are significantly higher than those of the females. Transfer functions and cross-correlations between arterial blood and CSF flow reveal significant age-dependence of phase-shift between these, as do the waveforms of arterial blood, as well as cervical-spinal and aqueductal CSF flows. These findings accentuate the need for age- and sex-matched control groups for the evaluation of cerebral pathologies such as hydrocephalus.

## Introduction

Analysis of cerebral arterial blood flow and its coupling with cerebrospinal fluid (CSF) dynamics can be of significant value for the diagnosis of pathologies such as hydrocephalus, provided that appropriate physiological reference standards exist. Age-related changes in cerebral and cerebrovascular physiology necessitate different reference standards for different age groups. In the present study on phase-contrast magnetic resonance imaging (MRI) derived data from twenty-two age and sex matched healthy subjects we show that age significantly influences various components of cerebral arterial blood flow and CSF dynamics. The novelty of the work at hand derives from a balanced study population, high temporal resolution MR imaging and a multi-tier data analysis which, in combination, allowed for the detection of significant differences in flow frequencies between the age groups that have, to our knowledge, not been published to date.

Various studies thus far have evaluated phase-contrast MRI flow measurements to assess arterial and venous blood flow, CSF flow in the aqueduct and subarachnoid space, and their mechanical coupling. Aging effects of CSF flow only in the aqueduct of healthy volunteers were studied by [1], [2]. Correspondingly, arterial and venous blood flows, as well as cervical spinal CSF flow were analyzed by Uftring et al [3] that accomplished nonparametric identification of transfer functions. In addition to the compartments analyzed by Uftring and co-workers, Stoquart-ElSankari et al [4] also assessed aqueductal CSF flow, investigating parameters such as mean and peak flows, their latencies and stroke volumes of CSF.

Feature points of the arterial waveforms, such as flow at aortic valve closure or maximum systolic flow of internal carotid and vertebral arteries of young healthy volunteers were evaluated in [5]. Hoi et al [6] investigated such feature points of the common, internal and external carotid arterial flow waveforms of elderly subjects and compared the findings to the results of Ford et al [5].

Several studies have compared generally elderly normal pressure hydrocephalus (NPH), asymptomatic ventricular dilation (VD), or Alzheimer's disease (AD) patients to healthy subjects of various age groups. CSF flow in the aqueduct was analyzed with regard to frequency components [7], [8], stroke volume [9], [10], and average flow rate [11]. Reconstructed instantaneous transcranial blood flow and interactions with the brain and CSF were described by waveform characteristics and transfer functions [3], [12]–[14]. Reconstructed transcranial blood flow was obtained by scaling measurable venous flow to match arterial blood volumes over one cardiac cycle. The age range, the female-to-male ratio, or the sex of the control groups often did not match the explored patient groups [14]–[16], while in other cases only the number but not sex of the control subjects was specified [11].

The study at hand quantifies age and sex related differences in physiological arterial blood flow and in cervical spinal and aqueductal CSF flows in a well-balanced population of volunteers. In contrast to previous studies, it addresses the pitfalls of flow signal processing and demonstrates the importance of data normalization for the purpose of meaningful comparison.

## Materials and Methods

### Ethics Statement

The investigations described herein were approved by the ethics committee of the Canton of Zurich and conducted according to the principles expressed in the Declaration of Helsinki. All subjects gave written informed consent.

### Participants

Two categories of healthy subjects were included in the study: Eleven young volunteers (5 female, 6 male), mainly students, were found via an online search portal. Eleven elderly volunteers (6 female, 5 male) answered an advertisement to retired employees of ETH Zurich. Thus, retired ETH Zurich employees of various professions, their relatives or acquaintances participated. The young group's subjects' age ranged from 21 to 29 years and the elderly group's from 64 to 81 years (Table 1). All participants drank 2 dl of a non-alcoholic beverage one hour prior to the measurements to present with comparable hydration levels. Next to the subjects' sex and age, body mass index (BMI) and height were recorded for statistical analysis. No further parameters such as brain size were considered.

**Table 1:** Mean and standard deviation (SD) of subjects' age, body mass index (BMI) and height, as well as aqueductal stroke volume [101] and average flow rate [11], (1).

	young volunteers (mean 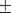 SD)						elderly volunteers (mean 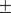 SD)					
Group (number)	all(11)		female(5)		male(6)		all(11)		female(6)		male(5)	
Age (year)	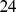	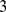	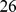	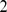	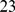	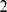	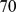	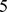	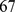	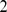	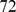	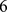
BMI (kg/m 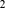 )	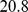	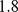	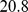	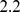	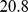	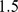	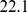	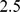	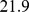	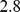	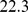	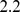
Height (cm)	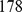	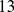	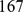	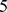	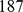	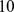	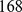	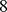	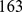	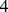	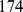	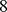
aqueductal stroke cvolume* ( 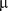 L)	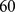	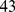	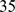	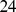	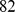	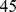	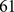	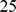	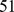	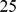	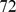	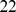
aqueductal avg flow** (mL/min)	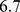	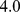	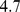	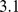	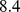	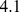	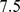	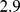	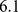	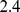	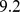	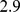
spinal avg flow (mL/min)	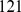	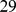	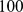	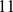	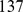	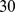	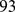	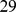	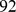	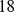	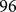	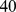
arterial avg syst flow (mL/min)	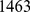	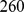	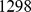	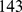	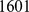	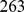	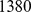	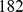	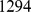	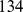	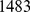	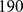

The average (avg) spinal CSF and systolic (syst) arterial flows used to normalize the data are listed. Significant differences and correlations are summarized below.

Difference between female and male volunteers:

* P = 0.033;

** P = 0.028.

Correlation in entire population of height with age 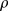 = 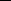
0.44, P = 0.04; and sex 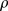 = 0.75, P
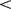
0.0001.

Correlation of height with sex in young group 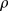 = 0.87, P = 0.0006; and in elderly group 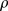 = 0.70, P = 0.017.

### Data Acquisition

All subjects were scanned in supine position on a 3T Philips Achieva MRI system (Philips Healthcare, Best, The Netherlands). Velocity measurements of blood flow in the common carotid and vertebral arteries, and of CSF flow in the cervical spinal canal and in the aqueduct were acquired using 2D cine phase-contrast gradient echo sequences.

A 3D time-of-flight angiography was used to plan the imaging slice of the arterial blood velocity measurement, which was acquired through-plane, 2 cm below the right carotid bifurcation. This imaging plane was additionally aligned perpendicular to the cervical spinal cord and covered the entire cervix. The signal was acquired using two small circular surface coil elements with a diameter of 7.5 cm placed next to the left and right carotid arteries, respectively. Imaging parameters for the velocity measurements in the common carotid and vertebral arteries were set to an in-plane acquisition resolution of 0.8×0.7 mm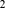 using a 176×175 acquisition matrix, 5 mm slice thickness, 2 signal averages, 100 cm/s encoding velocity (150 cm/s for one subject), and repetition time (TR)/echo time (TE) of 8.0/3.6 ms, which results in a minimal temporal resolution of 16 ms. All scans were retrospectively triggered with electrocardiographic (ECG) gating. The number of heart phases acquired was adapted to the cardiac frequency such that the number of time frames reconstructed equaled the number of heart phases measured (40 to 64 heart phases).

CSF velocity in the cervical spinal canal was acquired between the third (C3) and fourth (C4) cervical spinal vertebrae orthogonal to the spinal cord. The transversal imaging slice was positioned on a sagittal survey view. Imaging parameters were identical to those described for arterial flow measurements, except for the encoding velocity, which was set to 12 cm/s (15 cm/s for one subject). The resulting TR/TE were 9.4/5 ms, yielding a minimal temporal resolution of 18.8 ms (36 to 64 heart phases).

To acquire the aqueductal CSF velocity data, a sagittal, balanced gradient echo scan was used to plan the imaging slice. CSF flow was acquired through-plane in the central part of the aqueduct, using an eight-element head coil. The imaging parameters were in-plane resolution of 0.4×0.4 mm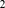 using a 248×250 acquisition matrix and fold-over suppression, slice thickness of 4 mm, 2 signal averages, encoding velocity of 15 cm/s, and TR/TE of 15/7.1 ms, which results in a minimal temporal resolution of 30 ms (22 to 46 heart phases).

### Data Processing

All data processing was performed semi-automatically in MATLAB R2009a (The MathWorks, Inc., Natick, MA, USA). Aliased pixels were detected with a jump tolerance of 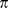 and corrected automatically with the ‘unwrap’ function implemented in MATLAB.

Otsu's method [17] is generally used to automatically perform histogram shape-based image thresholding to reduce a gray-level image to a binary image. The cross-sections of the arterial vessels and the aqueduct were segmented by means of the respective image masks. Therefore, one of the frames of the phase series acquired by MRI, which contained maximum velocities, was chosen and the region of interest (ROI) delimited with a polygon. Its area was segmented by means of the corresponding magnitude image contrast. If due to differences between the magnitude and the phase images the individual space was not adequately picked, the initial threshold level of Otsu's method was iteratively adjusted. The arteries were segmented frame by frame in order to account for the temporal change in their cross-section due to blood pulsation. The aqueduct and the cervical spinal canal were segmented in single-image frames, since their cross-sections vary only moderately over time. Due to the magnitude images' low contrast in the surroundings of the spinal canal, discrete Fourier transform was employed to segment the cervical spinal canal. The frequency content of all phase image frames in the preselected area of each voxel was calculated [18], and the respective frequency threshold was determined. The associated mask was drawn onto the selected image frame with the highest velocity content. The correctness of the semiautomatically segmented areas was verified over the entire cardiac cycle (CC). The velocity content of two one-pixel wide rims adjacent to the outside and inside of the cross-sections picked semiautomatically was compared to the evaluated velocity of the corresponding ROI. For any remaining velocity fractions in the neighboring outside area or lacking velocity fractions in the inside rim the respective threshold was adjusted and the semiautomatic segmentation was repeated.

The background phase error correction was initiated by choosing a preferably large rectangular area in the closest possible proximity of the ROI (e.g. the carotid arteries or the aqueduct) while avoiding overlap with it. The velocity content of the rectangular region was then checked manually for remaining velocity fractions. The background phase error was corrected by subtracting the mean velocity of this reference area. This procedure worked well for the arterial and aqueductal background phase correction. However, for the cervical spinal area, no rectangular area in vicinity of the spinal canal showed non-negligible velocity fractions. Instead, a one-voxel wide contour adjacent to the spinal canal was chosen and fulfilled the requirement that only minor velocity fractions were contained. The mean offset value was subsequently calculated and subtracted from the respective velocity data in the ROI [19].

Finally, the volumetric flow rate was obtained by summation of the products of area and respective velocity of all pixels included in the segmentation mask.

### Data Analysis

#### Normalization

Spinal and aqueductal CSF flows were normalized by the average flow rate of the corresponding CC. The average flow rate (1) is defined as the average of the absolute flow rates in caudocranial and craniocaudal direction within one cardiac cycle [11].
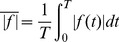
(1)


It does not correspond to the net flow rate as a result of CSF production.
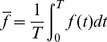
(2)


Arterial blood flow was normalized by the average flow rate of the systolic phase only. This approach was chosen as the arterial systole has a relatively constant duration, while the diastolic phase is dependent on heart rate [20]. In contrast, the waveforms of the CSF compartments show only small dependence on systole and diastole. The spatial normalization aims at accentuating flow curve patterns rather than analyzing amplitudes of volumetric flow rates.

Based on the various CC durations of the participants, the spacing of the frequency components is non-uniform and, therefore, the evaluated frequencies would not match (Appendix S1). Accordingly, analyzing frequencies as a mean of the data of several subjects requires the data to be previously scaled to identical periods. Truncating the waveforms with a rectangular window at the time length of the shortest dataset induced unwanted steps in the rest of the datasets and thereby caused the appearance of novel frequency components that were not present in the original data. Another approach with a more sophisticated window, such as a Hanning or a Kaiser window [21] considerably influenced the spectra. A stretching or compression of the waveforms relatively to obtain identical periods also proved inadequate: This approach neglected that while the length of systole remains fairly constant with change of heart rate, diastole is variable [20] and, therefore, altered the amplitudes of various frequency components markedly. The best and least distorting procedure to temporally normalize the flow curves consisted of leaving the different waveforms unchanged, and extending the diastolic period by a linear extrapolation of the last data point back to the value of the first point or reducing the end diastolic part to the nominal length. This approach is consistent with Hoi et al [6] who reported that “When it is desired to scale the characteristic waveforms to accommodate a different heart rate, it is recommended to extend or reduce the timing of the diastolic tail in order to preserve the timing of systole, rather than stretch or compress the entire waveforms.” The influence of this implementation is shown analytically in Appendix S1.

A nominal CC length of 1 s was chosen for the following reason: All but one CC in the study had a length of less than or equal to 1 s (mean 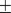 SD: 831
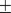
169 ms). Consequently, only one subjects' recording had to be shortened. The systolic periods were thus preserved, the CC lengths were homogenized and the amplitudes of the frequency components uniformly scaled. Finally, the data were resampled with an interval of 10 ms in order to get the same temporal discretization in all subjects. All subsequent data processing was performed with the scaled and resampled waveforms.

#### Feature points and characteristic waveforms

Volumetric arterial and CSF flow rates were analyzed with regard to their characteristic waveforms and feature points. The mean and standard deviation (SD) of the detected points were analyzed with respect to amplitude and timing of each age and sex group. Subsequently, the curves were fitted with a piecewise cubic Hermite interpolating polynomial [22]. In the aqueduct, average CSF flow rate [11] and stroke volume [10] were derived from raw data (Table 1). Stroke volume is defined as mean of the absolute values of the areas under the volumetric flow rate curves in systole and diastole.

#### Frequency analysis

The normalized data were analyzed by discrete Fourier transformation [23]. Individual frequencies obtained through this analysis are referred to as frequency components in the subsequent text and expressed in Hertz (Hz). The sequences' noise levels were determined using the same image section as applied in the segmentation step for background phase error correction. To assess the noise level, we considered a synthetic signal which consisted of a sinusoidal wave distorted by white noise (mean 0 and variance 1). The overall frequencies were analyzed by stepwise reduction of the amplitude of the sine wave from 1 to 0. The frequency component of the sinusoidal wave with amplitude of one-fifth SD of the white noise was correctly specifiable from the noise frequency components. Subsequently, one-fifth SD of every subject's background noise per compartment (arteries, spinal canal and aqueduct) was computed, and the overall maximum of all results per compartment was taken as frequency threshold. In neither compartment was the Nyquist frequency a limiting factor. As a result of the normalization by the subject's average volumetric flow rates, the zero frequency no longer corresponded to net flow and was thus not included in the analysis.

#### Transfer function

Transfer functions (TF) were evaluated by a time-invariant black-box system identification approach [24], which allowed identification of the complex cranial system without any prior assumptions about its structure. This approach is purely data-driven. In the present study, the prediction-error identification method (PEM) that yields causal system models was applied to the input-output data of one CC. A linear fifth-order model proved sufficient to capture all the relevant dynamics in either compartment (see Appendix S1 for details).

#### Cross-correlation

Temporal relationships of the pulse transmission from the arterial inflow to both CSF flows were analyzed by cross-correlations [24]. With the corresponding results, the delays of peak caudal aqueductal and cervical spinal CSF flows relative to the peak arterial flow were computed.

### Statistical Analysis

Non-normally distributed data were logarithmically transformed before analysis. Characteristic waveforms, frequencies, transfer functions, and cross-correlations of the young and elderly age groups were evaluated using repeated measures analysis of variance (ANOVA) with Greenhouse-Geisser correction to compensate for non-sphericity. The ANOVA was performed within both age groups and between the data of the young and elderly participants. Feature points of the waveforms, individual frequency components and delays were compared with a nonparametric Mann-Whitney test. Correlations were calculated with Spearman's rho. All data were analyzed using SPSS Statistics 17.0 (SPSS Inc., Chicago, IL, US). Differences were considered significant at P
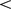
0.05. All results are tabulated or plotted as mean 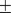 SD.

## Results

The Mann-Whitney test of the female and the male volunteers' stroke volume and average flow in the aqueduct (Table 1) show a significant difference with P = 0.033 and P = 0.028, respectively. In contrast, there is no significant age dependence of stroke volume and average flow in the aqueduct.

The body height of all female participants is below the average of 173 cm and that of all but one male participant above 173 cm. Height correlates with age (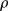 = 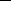
0.44, P = 0.04) and with sex (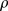 = 0.75, P
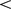
0.0001). Within the respective age category, height correlates with sex: 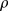 = 0.87 and P = 0.0006 in the young group, and 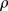 = 0.70 and P = 0.017 in the elderly group. However, height does not correlate with stroke volume or average flow in the aqueduct.

### Characteristics of waveforms

**Figure 1:**
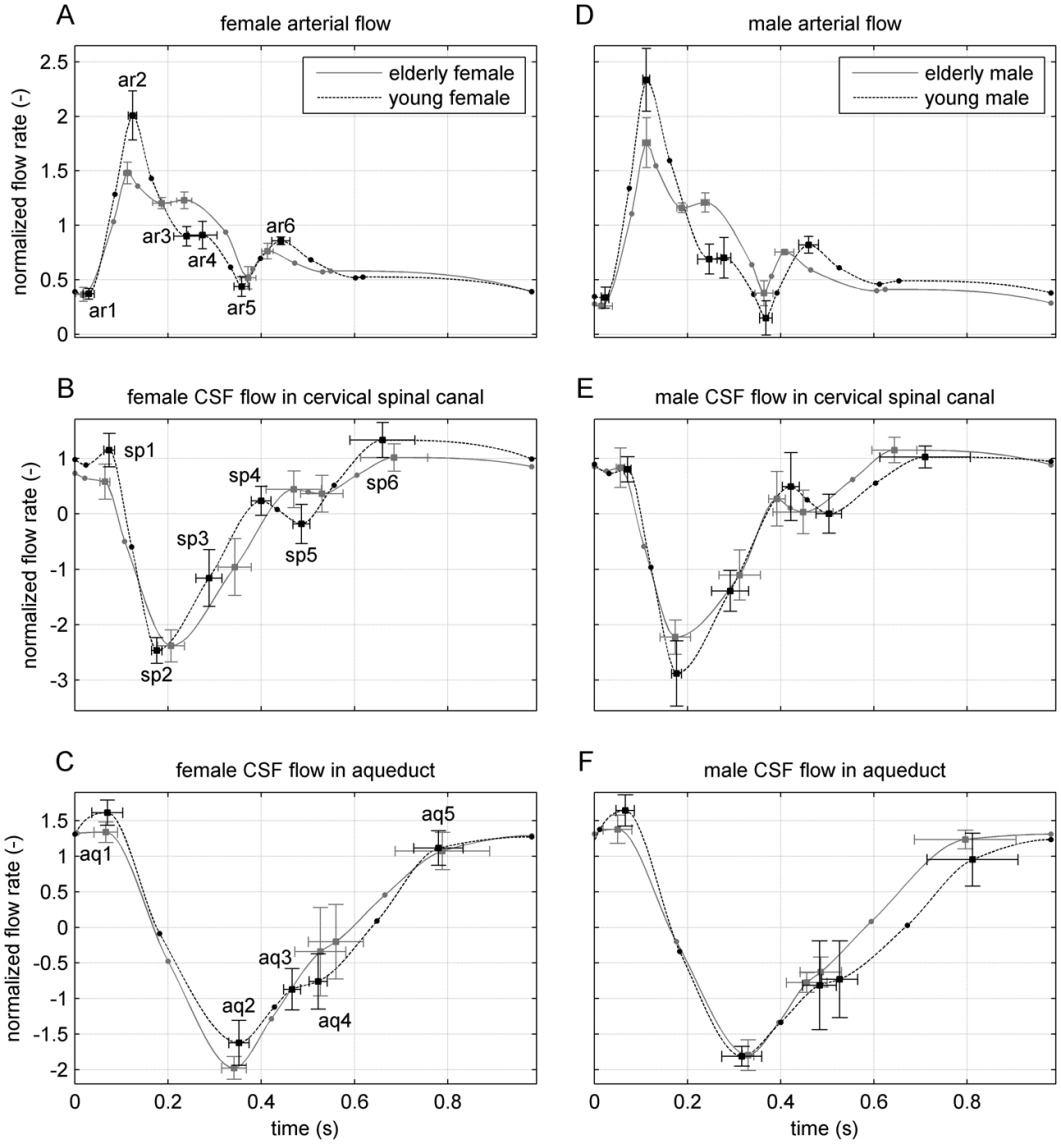
Feature points with corresponding standard deviation (SD) error bars and piecewise cubic Hermite interpolated polynomial fits [**22**] of arterial and cerebrospinal fluid (CSF) flow in the elderly (gray line) and young groups (dashed black line) as a function of time. The left column **A**–**C** shows results for females, right for males **D**–**F**. Top row: Sum of mean (over subjects) normalized flow velocity curves in the left and right common carotid arteries and the left and right vertebral arteries. Middle row: Mean CSF flow in the cervical spinal canal at the level of C3–C4 (between the third and fourth vertebrae). Positive values correspond to flow in cranial direction. Bottom row: Mean CSF flow in the aqueduct (positive values: cranial flow). In panels **A** and **D**, the feature points are labeled ar1–ar6, in **B** and **E** sp1–sp6 and in **C** and **F** aq1–aq5 (Table 2).

Differences of waveform characteristics between the data of the young and elderly subjects were analyzed using repeated measures ANOVA. Amplitudes (P = 0.0095) and temporal locations (P
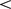
0.0001) of the young subjects' arterial flow feature points are significantly different from those of the elderly subjects, Figs. 1A and 1D. The cervical spinal, Figs. 1B and 1E, and aqueductal CSF flow, Figs. 1C and 1F, amplitudes and timings of the two age groups do not show any significant differences. In the young age group, the arterial flow features depend significantly on sex (P = 0.018), while in the elderly group only the timing of the cervical spinal flow feature points differs significantly (P = 0.013) between sexes. In both age categories, there is no sex dependence of arterial flow timing, spinal flow amplitude, and aqueductal flow amplitude and timing.

The Mann-Whitney analysis of the feature points (Table 2) of the flow curves in relation to age shows the following results: The maximum of mean (over subjects) volumetric flow rates in the common and vertebral arteries, Figs. 1A and 1D, feature point ar2, is significantly higher in the young subjects compared to the elderly (P = 0.0006). In general, elderly volunteers have a higher and more distinct late systolic peak (P
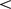
0.0001, ar4), a higher flow rate at aortic valve closure (P = 0.045, ar5), and a reduced flow rate at the diastolic maximum (P = 0.0095, ar6) than the young. There are significant timing differences between the young and elderly groups' feature points at ar3, ar4 and ar6 (all P = 0.0003). The maximum of the mean caudal flow rate of the spinal CSF of the young group is not significantly higher, but the pulse is narrower (P = 0.018) than that of the elderly, Figs. 1B and 1E, sp3. The results of the young group show a considerable local minimum at sp5 (P = 0.053), which can also be noticed in the aqueductal flow, Figs. 1C and 1F, aq4. The progression of the aqueductal flow of the elderly group is smoother, and the maximum cranial flow rate is smaller (P = 0.0064) than that of the young group.

**Table 2:** Legend of waveform feature points (Fig. 1) in the corresponding compartments.

arterial blood flow		cervical spinal CSF flow		aqueductal CSF flow	
ar1	diastolic minimum	sp1	local maximum cranial flow	aq1	maximum cranial flow
ar2	systolic maximum	sp2	maximum caudal flow	aq2	maximum caudal flow
ar3	late systolic local minimum	sp3	inflection point	aq3	first inflection point
ar4	late systolic peak	sp4	local maximum	aq4	second inflection point
ar5	minimum at aortic valve closure	sp5	local minimum	aq5	third inflection point
ar6	diastolic maximum	sp6	maximum cranial flow		

Several feature points of the flow rates in the three compartments correlate with body height: Of the arterial flow, the magnitudes of the maximum flow (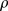 = 0.68, P = 0.0006), the late systolic local minimum (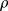 = 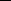
0.70, P = 0.0003), the late systolic peak and the aortic valve closure (both 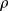 = 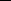
0.68, P = 0.0005), as well as the timing of the late systolic peak (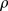 = 0.47, P = 0.026) correlate with height, as do the timing of the cervical spinal pulse width (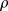 = 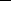
0.45, P = 0.035) and the amplitude of the aqueductal cranial flow (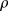 = 0.44, P = 0.042).

### Frequency Analysis

**Figure 2:**
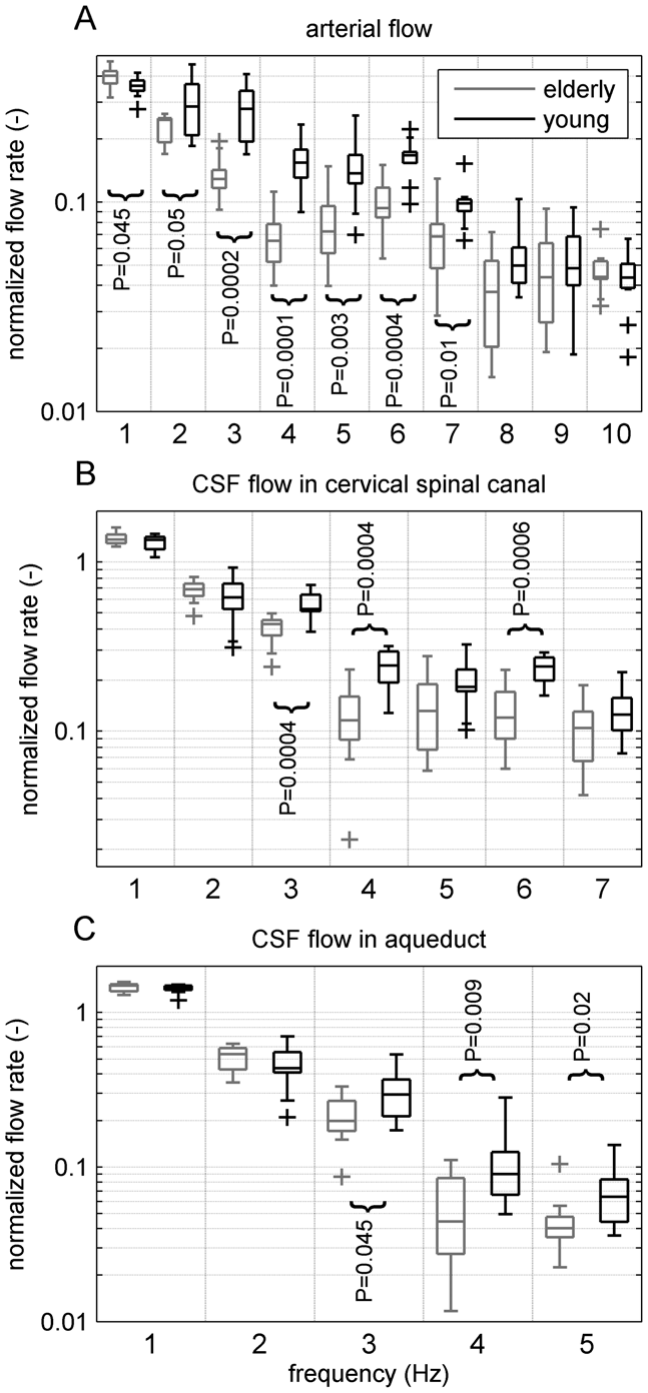
Juxtaposed frequency components (Hz) of flow in the elderly (gray, left) and young groups (black, right) illustrated using Boxplots. The upper and lower edges of each box represent the 25th and 75th percentiles, respectively. The center line represents the median and the whiskers extend to the most extreme data points not considered outliers, which are plotted individually (+). In each subfigure, frequency components are separated by dotted lines. In **A**, the first to the 10th frequency components of the combined flow rates in the carotid and the vertebral arteries are plotted, in **B**, the first to the seventh frequency components of the cerebrospinal fluid (CSF) flow rate in the cervical spinal canal at the level of C3–C4 and in **C**, the first to the 5th frequency components of CSF flow rate in the aqueduct. Curly brackets indicate significant differences of the respective frequency component's magnitudes detected with the Mann-Whitney test. Note that the fourth frequency components of the arterial flows **A** hardly overlap, thus the corresponding asymptotic 2-tailed p-value is 0.0001.

Intra- and inter-subjects effects were evaluated by repeated measures ANOVA. The frequency components of the arterial flow as well as of the CSF flow in the spinal canal and in the aqueduct are found to be significantly different within the age groups with P
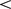
0.0001 (Fig. 2). The differences between the young compared with those of the elderly volunteers' frequency components in Figs. 2A–2C are significant with P = 0.0009, P = 0.0005 and P = 0.0076. Frequency components of the elderly group's flow rate (gray, left) are juxtaposed to those of the young group (black, right). Significant individual frequency components evaluated with the Mann-Whitney test are marked with curly brackets and the respective p-values are indicated, Figs. 2A–2C. In the elderly group, the median magnitude of only the fundamental frequency of the arterial flow is higher than the respective median in the young group, Fig. 2A. Above 1 Hz up to 9 Hz, all median magnitudes of arterial flow frequency components of the young group are higher than those of the elderly. In the CSF space, Figs. 2B and 2C, the magnitudes at 1 and 2 Hz are comparable. Beyond 2 Hz, the median magnitudes of the young subjects are higher than those of the elderly.

### Transfer Functions

**Figure 3:**
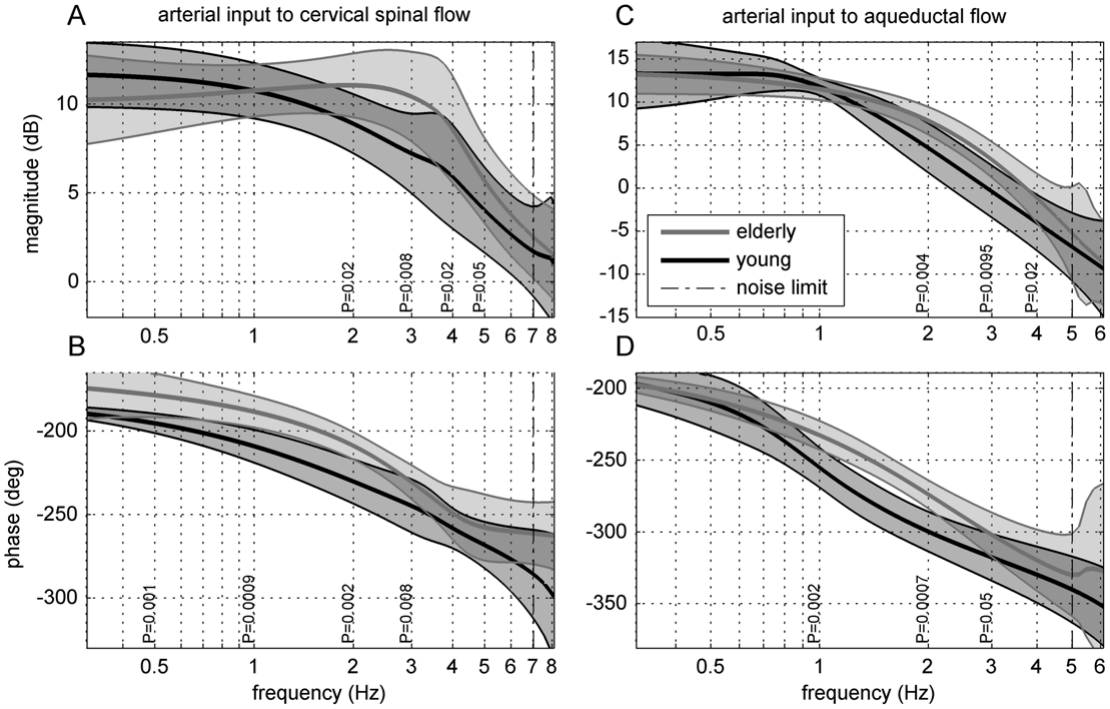
Mean magnitude and phase of transfer function (bold line) and standard deviation (gray shading) from arterial input to cervical spinal cerebrospinal fluid (CSF) flow, A and B, at the level of C3–C4, and to aqueductal CSF flow, C and D, of elderly (gray line) and young (black line) groups as a function of frequencies. Significant differences of the magnitude and phase as detected by the Mann-Whitney test are depicted at the respective frequency. At 2 Hz the phases of the arterial-to-aqueductal flows hardly overlap **D**, resulting in an asymptotic 2-tailed p-value of 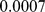.

Transfer functions of the arterial input to the cervical spinal as well as to the aqueductal CSF output are represented by their magnitudes and phase angles versus frequency in Fig. 3. Magnitudes indicate the gain at the respective frequency, whereas phases represent transmission lag. The frequency axes are plotted in logarithmic scale. Values at frequencies beyond the indicated noise limit (vertical dash-dot line) are considered negligible, as described in the Methods section under Data Analysis. At 1 Hz, the mean magnitudes of both the young and elderly subjects are equal, Figs. 3A and 3C. The two magnitudes then diverge up to approximately 3 and 2 Hz, respectively, and converge again at higher frequencies. The corresponding p-values are indicated in Fig. 3. Above 0.5 Hz, the mean phases of the elderly group in either compartment are smaller than those of the young group, Figs. 3B and 3D. Phase lag differences between the two groups are highest at 1 Hz in the transmission of the arterial-to-cervical spinal flow and at 2 Hz in the transfer of the arterial-to-aqueductal CSF flow.

Repeated measures ANOVA between the two age groups was evaluated at every fifth data point from 0.5 Hz up to 7 Hz in the case of the arterial-to-cervical spinal CSF transfer function and up to 5 Hz in the transfer function to the aqueduct. The phases of arterial-to-cervical spinal CSF flows are significantly different (P = 0.0004), Fig. 3B, as are the magnitudes and phases of the arterial-to-aqueductal CSF flows (P = 0.024 and P = 0.0027), Figs. 3C and 3D. The mean magnitude of either transfer function shows low-pass characteristics, Figs. 3A and 3C. The mean crossover frequency of the arterial transfer to spinal flow of the young group is approximately 0.5 Hz and of the elderly group 3 Hz. The subsequent magnitude decrease of the elderly group is steeper, indicating a second-order behavior. In the transfer of the arterial-to-aqueductal flow, the mean crossover frequencies of the young and of the elderly groups are 0.8 Hz and 0.5 Hz, respectively.

### Cross-correlation

**Figure 4:**
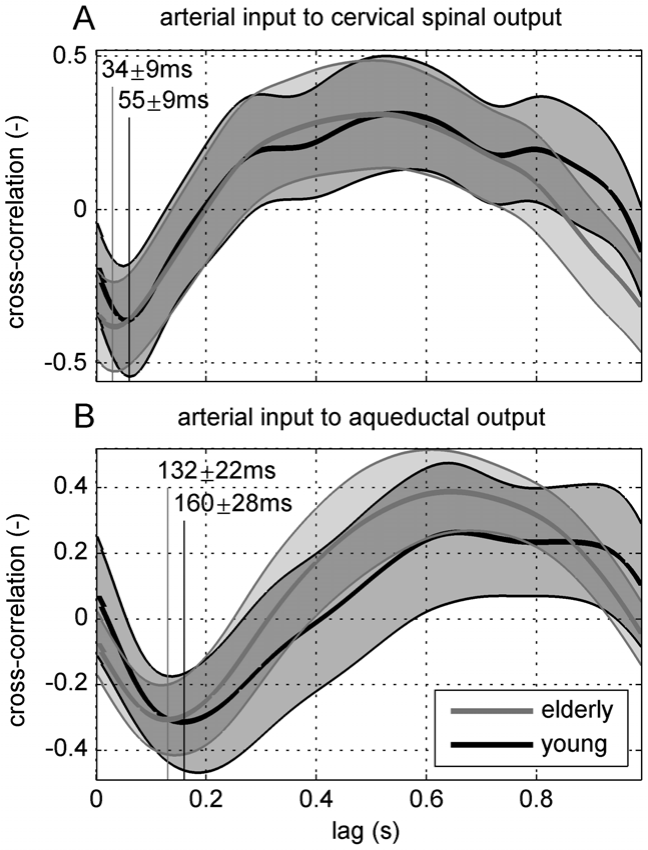
Cross-correlation of arterial input to cervical spinal A (at the level of C3–C4) and to aqueductal B cerebrospinal fluid flow, mean (bold line) and standard deviation (gray shading) of elderly (gray line) and young (black line) groups' results. The vertical lines depict the mean delays from the maximum systolic peak flows to the maximum caudal flows in either CSF compartment.

Transmission latencies from the arterial input to the spinal and the aqueductal CSF compartments were determined via cross-correlation. The maximum caudal cervical spinal CSF flow latency shows a significant difference (P = 0.0004) between the elderly subjects with a delay of 34
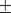
9 ms and of 55
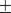
9 ms for the young group, Fig. 4A. Additionally, a significant difference (P = 0.019) was found between the delay of the maximum caudal aqueductal flow of 132
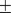
22 ms for the elderly and of 160
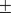
28 ms for the young group, Fig. 4B. In either plot, the mean cross-correlation curve of the elderly is smoother than that of the young group, which is linked to the higher frequency content measured within the group of young adults, Fig. 2. The inter-curve difference of the two age groups was tested at every fifth lag, that is every 50 ms from 0–950 ms. However, aside from the mentioned delays, there were no other significant age-related differences.

## Discussion

Our results demonstrate that age significantly influences various characteristics of arterial blood flow into the brain and its coupling with the dynamics of the cerebrospinal fluid. Concretely, transfer functions of arterial input to both cervical spinal and aqueductal CSF output distinctly characterize the here studied young and elderly age groups. Corresponding differences in phase lag are confirmed by cross-correlation of the arterial input and the respective output. We further show that significant differences exist between the male and female subgroups in arterial flow waveforms, which indicate that reference standards for diagnostic purposes should not only be age, but also sex specific. In the paragraphs below, we first outline limitations of our study, and then discuss individual aspects of our results in comparison to data reported in the literature.

This study has three main limitations. First, the number of studied subjects is comparably small. It is conceivable that a larger study population may reveal further significant differences that could not be recognized with the current population size. Second, the type of beverage consumed by the participants to reach comparable hydration levels was not controlled for. Caffeine, for example, is known to affect cerebral blood flow [25], [26] and CSF production [27]. Third, while arterial inflow, cervical spinal and aqueductal CSF flows were measured, venous outflow was not. This is due to that fact that even though jugular veins often appeared large in our subjects' MR images, the effectively detectable blood flow through them was frequently negligible in comparison to the arterial inflow to the cranium. This is a direct result of the existence of a large number of different venous drainage pathways besides the jugular veins, as described by several authors [28]–[30]. With the distribution of venous drainage over various vessels of dissimilar sizes and disparate flow rates, the quantification of venous flow via MRI becomes challenging to a degree where one cannot guarantee its reliability. We are aware that other groups who use the same MRI methods have come to a different conclusion regarding the reliability of venous flow quantification [13], [14].

Frequency analyses of cervical spinal and aqueductal flows allow for the identification of age effects that cannot be observed in the time domain. For instance, the elderly group's aqueductal flows resemble sinusoidal waves with gentle slopes, whereas the curves of the young group show distinct inflection points. Most magnitudes of the frequency components in all three compartments differ considerably between the two age groups and thus accentuate the various characteristics of the age groups' waveforms.

In either age group and for all subjects, arterial inflow to both the spinal and the aqueductal flow transfer functions show low-pass characteristics. Above 0.5 Hz, the two mean transmissions of the elderly group lead in phase those of the young group. De Marco et al [15] analyzed arterial-to-aqueductal TFs of young volunteers and elderly patients with VD and NPH. The magnitudes of the second to the fifth frequency components of their elderly patients were higher than those of their young healthy volunteers. This outcome is comparable to our results at the corresponding frequencies: Elderly volunteers have higher magnitudes than the young ones.

We confirmed phase differences obtained through the investigation of transfer functions by analysis of corresponding cross-correlations of arterial input and respective cerebrospinal fluid flow output, which is a data-driven, phenomenological approach free of assumptions. The delays observed in the elderly group are significantly shorter in both transmissions to the aqueduct and the spinal canal. We interpret these shorter delays as a result of the faster wave propagations in elderly subjects due to their stiffer arterial walls [31].

14 healthy volunteers in our study, 10 of which male subjects, have stroke volumes in the aqueduct greater than 42 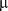
L, which is a commonly used threshold value for clinical assessment of shunt responsiveness based on [10]. These results, together with earlier data of ours [32], suggest that this threshold value should be reevaluated. The average flow rates of all participants, which do not correspond to CSF production rate in the third and lateral ventricles, are below 18 mL/min: Values above this limit are seen by Luetmer et al [11] as indicative of NPH. Both stroke volume and average flow rate are significantly sex dependent (male higher than female), but not age dependent, although a trend towards higher values in the elderly is visible for both parameters. This outcome contrasts with the corresponding values in the study by Stoquart-ElSankari et al [4], where average flow rates and stroke volumes of young volunteers were higher than those of the elderly. Their male-to-female ratios of the young and the elderly subjects were 16∶3 and 4∶8, respectively. The male bias of the former group may have caused an overestimation of the young group's flow rates and stroke volumes, while underestimating the values of the elderly. The sex dependence of aqueductal stroke volume and average flow rate have thus to be taken into account in the design of studies that investigate these parameters, e.g. by a balanced ratio of female-to-male subjects or, where applicable, through a sex matched control group.

Various research groups have analyzed arterial, cervical spinal, and aqueductal CSF volumetric flow rates. Some assessed flow as a percentage of one CC [4], while others plotted flow versus the cardiac phases (CPh) acquired by MRI [14], [15] or as we did, versus time [6], [12]. As a consequence of the different evaluation methods used in the various studies, the respective results cannot be compared. Evaluating measurements versus time as done in the current study takes into account the rather constant systolic phase and the heart-rate-dependent diastolic period. In contrast, analyzing data as a percentage of the CC or against CPh stretches or compresses the waveforms, disregarding physiological constraints. With respect to discrete Fourier analysis, the subject-dependent CC durations cause non-uniform spacing of the frequency components. Accordingly, analyzing frequencies as a mean of the data of several subjects requires the data to be previously scaled to identical periods. Our approach of prolonging or cutting the diastolic phase and leaving the systolic phase entirely unchanged both preserves physiological constraints and, at the same time, provides identical periods that are necessary for frequency analysis.

Our data show that the arterial waveform shapes of the elderly group differ significantly from those of the young group. The maximum volumetric flow rate of the young and the late systolic peak of the elderly group are distinctively higher than in the respective other group. The observed high late systolic peak of the elderly subjects can be explained by their stiffer arterial walls that cause higher pulsation amplitudes than in the softer arteries of younger subjects and faster pressure wave reflections from distal boundaries, such as capillaries, where impedance changes markedly [31], [33].

In the young group, the pulse of the cervical spinal flow is narrower, i.e. the duration of the caudal cervical spinal CSF flow is shorter. Stoquart-ElSankari et al (2007) described a comparable finding as “a loss of sharpness in the cervical CSF curve peaks in the elderly group.” The wider cervical spinal CSF waveforms of our elderly group can be explained by the proximity of the respective anatomic structures to the arterial input and the corresponding wider arterial waveforms due to their earlier pulse wave reflections. The spinal waveforms of our young subjects show distinct local maxima due to aortic valve closure, which is also noticeable, though to a lesser degree, in their aqueductal flow. Balédent et al [14] communicated equivalent results in a comparison of cervical spinal characteristic waveforms between young healthy volunteers and rather elderly communicating hydrocephalus (CH) patients. They hypothesized that “alterations in CSF flow dynamics induced by age differences would be negligible compared with the large changes by CH.”

The normalized maximum cranial aqueductal flows of our elderly subjects are significantly lower than the youngs' and their waveforms are in the style of a sinusoidal wave. Softening of the elderly brain parenchyma as shown by multifrequency magnetic resonance elastography [34], and the concomitant decrease of parenchymal viscoelasticity may result in a lower resistance to distension [35], and may, therefore, dampen pulse transmission from the arterial input to the ventricular system in the elderly brain. This may explain the smoother and to a certain extent sine wave-like form of the aqueductal CSF flows we observed in the elderly group.

In contrast to Sack and co-workers [34], Czosnyka et al [36] suggest in their work on NPH patients an “increase of the brain's elastance coefficient and of the resistance to CSF outflow, indicating a stiffening of the brain with age.” Similarly, Uftring et al [3] report age-related changes of the coupling of vascular pulsations to the cervical spinal cord and CSF, stating that “[elderly subjects] had a tendency for vascular pulsations to cause relatively decreased cervical spinal CSF pulsations compared to the normal young adults, (…) which could be caused by increased rigidity of brain.” These seemingly contradictory statements can be reconciled if one considers that a softening of the brain parenchyma and a stiffening of the intracranial space as a whole are not mutually exclusive: Stiffening of the intracranial space with age is related to the stiffening of the arterial walls [31] and to an increased intracranial water content due to loss of brain tissue [37]. Softening of the brain parenchyma is a result of changes in brain tissue structure [38]. Thus depending on the definition of ‘stiffness’ or ‘compliance’, apparently different results can be obtained. In either case, the here reported age-dependence of arterial blood flow and its coupling with cerebrospinal fluid dynamics is consistent with the literature.

Vascular changes as discussed and brain atrophy are the most intuitive potential causes for the here observed differences between the young and elderly groups. According to Enzinger et al [37], neurologically asymptomatic elderly experience continuing brain volume loss that appears to accelerate with age. By comparing groups of elderly subjects with matched sex, age and level of cardiovascular disease, but difference in brain parenchymal fraction [39], one could test the hypothesis that atrophy is responsible for age related difference in intracranial dynamics. Conversely, cardiovascular disease as possible cause could be investigated.

We hypothesize that the observed sex dependence of aqueductal CSF stroke volume and average flow is a result of the probable size difference between the male and female volunteers' brains. While brain size was not measured in this study, it is well documented that the male brain is in average larger than the female brain [40], containing a larger ventricular system [41]. Consequently, a higher absolute ventricle volume variation during the cardiac cycle can be expected in the male, leading to an increased flow rate through the aqueduct. It cannot be excluded that sex dependence of hormone makeup, brain stiffness [34] and rate of brain atrophy [42] also play a role.

### Conclusion

Cerebral arterial inflow and cerebrospinal fluid dynamics, as well as their coupling, show significant age-dependence that can be discerned by frequency analysis of phase-contrast MRI data with sufficiently high temporal resolution and after proper temporal normalization. This suggests, together with the observed sex-dependence of the combined carotid and vertebral arterial waveform, that respective reference standards for the evaluation of cerebral pathologies such as hydrocephalus should be both age- and sex-specific.

## Supporting Information

Appendix S1 Details on nominal cardiac cycle length and transfer function identification.
(PDF)
