# Supplementary material for: Age-Specific Characteristics and Coupling of Cerebral Arterial Inflow and Cerebrospinal Fluid Dynamics
Source: PLoS One. 2012 May 30;7(5):e37502. doi: 10.1371/journal.pone.0037502 (PMC3364266; doi:10.1371/journal.pone.0037502)
Supplement: Appendix S1 — Details on nominal cardiac cycle length and transfer function identification. (PDF) [file pone.0037502.s001.pdf]

Appendix S1 to "Age-Specific Characteristics and Coupling of Cerebral Arterial Inflow and Cerebrospinal Fluid Dynamics"

Marianne Schmid Daners[1], Verena Knobloch[2], Michaela Soellinger[2,3], Peter Boesiger[2], Burkhardt Seifert[4], Lino Guzzella[1], and Vartan Kurtcuoglu[5,*]

1 Institute for Dynamic Systems and Control, Department of Mechanical and Process Engineering, ETH Zurich, Zurich, Switzerland, **2** Institute for Biomedical Engineering, University and ETH Zurich, Zurich, Switzerland, **3** Neuroimaging Research Unit, Department of Neurology, Medical University Graz, Austria, **4** Institute of Social and Preventive Medicine, University of Zurich, Zurich, Switzerland, **5** Laboratory of Thermodynamics in Emerging Technologies, Department of Mechanical and Process Engineering, ETH Zurich, Zurich, Switzerland
$*$ E-mail: vartan.kurtcuoglu@ethz.ch

Nominal cardiac cycle length

The influence of extending diastole to normalize the volunteers' cardiac cycle length can be shown via the example of a periodic square pulse wave [1] with pulse duration of 2φ and period of ωT, see (1) and Fig. A.

$$f(\omega t) = \frac{2h}{\pi}\Big(\frac{\varphi}{2} + \frac{sin(\varphi)}{1}\cos(\omega t) + \frac{sin(2\varphi)}{2}\cos(2\omega t) + + \frac{sin(3\varphi)}{3}\cos(3\omega t) + \cdots + \frac{sin(n\varphi)}{n}\cos(n\omega t)\Big) \quad (1)$$

$$\omega = \frac{2\pi}{T} \quad (2)$$

$$\varphi = \frac{\omega T_{sys}}{2} = \frac{\pi T_{sys}}{T} \quad (3)$$

This periodic square pulse can be rendered comparable to the temporal course of a subject's cardiac cycle by taking T_{sys} (systole) constant and T variable, corresponding to different CCs. An additional similarity between the real flow curves and the square pulse is the high signal amplitude during T_{sys} and the small amplitude during the rest of the period.

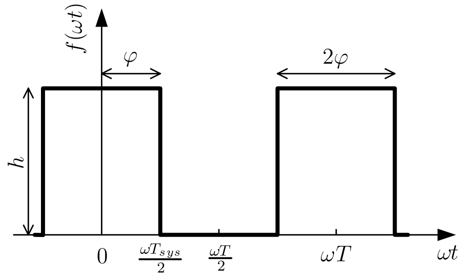

Figure A. Periodic square wave with pulse duration of $2\varphi = \omega T_{sys}$ and period length of ωT.

The flow curves are normalized by the average flow rate $\overline{|f|} = \frac{1}{T}\int_0^T |f(t)|\, dt$. The general form of thenormalized Fourier series is

$$f_N(\omega t) = \frac{f(\omega t)}{\overline{|f|}} \quad (4)$$

$$= a_0 + a_1\cos(\omega t) + a_2\cos(2\omega t) + + \cdots + a_n\cos(n\omega t), \quad (5)$$

where $a_0,\ a_1, \ldots,\ a_n$ are the Fourier coefficients. For the periodic square pulse wave $f(\omega t)$ (1) with $\overline{|f|} = \frac{hT_{sys}}{T}$, the resulting Fourier coefficients are

$$a_0 = \frac{2h\varphi}{2\pi}\overline{|f|}^{-1} = \frac{hT_{sys}}{T}\frac{T}{hT_{sys}} = 1 \quad (6)$$

$$a_1 = \frac{2h\sin(\varphi)}{\pi}\overline{|f|}^{-1} = \frac{2h}{\pi}\sin\big(\frac{\pi T_{sys}}{T}\big)\frac{T}{hT_{sys}}$$
$$= \frac{2T}{\pi T_{sys}}\sin\big(\frac{\pi T_{sys}}{T}\big) \quad (7)$$

$$a_2 = \frac{2h\sin(2\varphi)}{2\pi}\overline{|f|}^{-1} = \frac{2h}{2\pi}\sin\big(\frac{2\pi T_{sys}}{T}\big)\frac{T}{hT_{sys}}$$
$$= \frac{T}{\pi T_{sys}}\sin\big(\frac{2\pi T_{sys}}{T}\big) \quad (8)$$

$$\vdots$$

$$a_n = \frac{2T}{n\pi T_{sys}}\sin\big(\frac{n\pi T_{sys}}{T}\big) \quad (9)$$

The frequency component a_0 corresponds to the normalized net flow. In this special case, the non-normalized net flow is

$$a_0' = a_0\overline{|f|} = \overline{|f|} = \overline{f} \quad (10)$$

since for this particular periodic square pulse wave $|f(\omega t)| = f(\omega t)$. Fig. B depicts the dependence of the Fourier coefficients' amplitudes and their frequency spacings with respect to the variable time T.

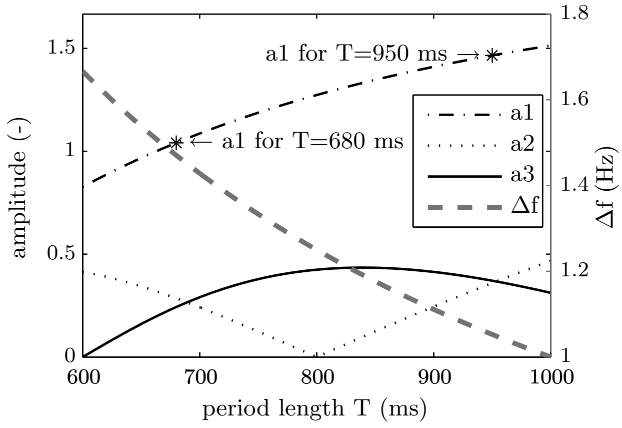

Figure B. Fourier coefficients of the normalized square wave (5) with a constant pulse width T_{sys} depend on the period length T, as do the spacings Δf of the frequency components.

We show 1) that the frequency components $a_1 \ldots a_n$ depend on the period length T (CC) as well as on the pulse width T_{sys} (systole); 2) that even if the systoles of two subjects are equal, but the CCs are not, the amplitudes of the frequency components will be different; and 3) the spacings of the frequency components depend on T. For these reasons, it is necessary to normalize the heart rates if frequencies are analyzed as a mean of several subjects.

The extension of the diastolic period has the effect that the amplitudes of the frequency components are scaled uniformly. This effect would also be observed if the same subject, instead of having his or her period extended, would be scanned at the corresponding lower heart rate. Additionally, frequency

patterns' characteristics reported in [2–4] are comparable to our homogenized and pooled results (Fig. 2 in main article), which indicates that the data were rendered comparable without changing their characteristics.

Transfer function identification

A parameter estimation method [5] was applied to the input-output data of one CC. The insufficient frequency content of the signal impeded the use of a nonparametric identification method, e.g. calculating the TF by dividing each amplitude of the output by the corresponding input frequency component, since it would result in an unduly noisy function where the magnitude and phase patterns would not be detectable. A linear fifth-order model was analyzed by the prediction-error identification method (PEM) which, using a least-squares estimation, identifies the model parameters by minimizing the difference between model output and measured data. The input-output data was first preprocessed by detrending, i.e. by the elimination of offsets and drifts. Subsequently, the influence of the input to the output was characterized without considering the actual data level. Flow measurements of one volunteer were repeated and the identified models validated with the data of the second scan. The models were simulated with the input from the respective validation data, while the corresponding outputs ($\hat{y}$) were compared to the measured data (y).

The fit was calculated according to (11), with $\overline{y}$ being the mean of the measured output.

$$fit = 100 * \left(\frac{1 - \|\hat{y} - y\|}{\|y - \overline{y}\|}\right) \tag{11}$$

The fit of the identified models for the arterial-to-cervical flow was 80% and for the arterial-to-aqueductal transmission 95%.

Nonlinear Hammerstein-Wiener and nonlinear ARX models were also tested, validated and compared to the linear models. The non-linear models gave a poorer fit than the linear ones and most identified non-linear models became instable during the validation process.

References

1. Bartsch H (1985) Taschenbuch Mathematischer Formeln. Fachbuchverlag Leipzig, 8. edition.

2. Frayne R, Rutt BK (1993) Frequency response to retrospectively gated phase-contrast mr imaging: effect of interpolation. J Magn Reson Imaging 3: 907–917.

3. Alperin N, Vikingstad EM, Gomez-Anson B, Levin DN (1996) Hemodynamically independent analysis of cerebrospinal fluid and brain motion observed with dynamic phase contrast MRI. Magn Reson Med 35: 741–754.

4. Thomsen C, Sthlberg F, Stubgaard M, Nordell B (1990) Fourier analysis of cerebrospinal fluid flow velocities: MR imaging study. the scandinavian flow group. Radiology 177: 659–665.

5. Ljung L (1999) System identification: theory for the user. Prentice Hall: Upper Saddle River, N.J., 2. edition.
